# Vertical HIV transmission in perinatally-exposed infants in South-Rift region of Kenya: a retrospective cross sectional study

**DOI:** 10.1186/s12889-017-4124-z

**Published:** 2017-02-17

**Authors:** Everline Ashiono, Dunstan Achwoka, Jamlick Mutugi, Joel Rakwar, Andrew Wafula, Otto Nzapfurundi Chabikuli

**Affiliations:** 1 FHI360, The Chancery, 2nd and 3rd Floor, Valley Road, Nairobi, Kenya; 2 FHI360, 2nd Floor, Block B, Hatfield Gardens, 333 Grosvenor Street, Hatfield, Pretoria, 0083 South Africa; 3 Sefako Makgatho Health Sciences University (SMU), P. O. Box 544, Medunsa, 0204 South Africa

**Keywords:** HIV, Early infant diagnosis, Prevention of Mother to Child Transmission, Africa

## Abstract

**Background:**

Despite proven efficacy of the prevention mother-to-child transmission of HIV strategy, its adoption in Africa has remained slow. In Kenya, its effectiveness remain unknown. The aim of this study was to assess the effectiveness of a prevention of mother-to-child transmission program in Kenya.

**Methods:**

This retrospective cross-sectional study analyzed 2,642 records of HIV-exposed infants who had a deoxyribonucleic acid polymerase chain reaction test done. The main outcome measure was HIV vertical transmission rates, stratified by i) infant age at diagnosis, ii) maternal prophylaxis and iii) infant mode of feeding. The characteristics of the infants who tested positive were compared to those who tested negative using Chi-square and Wilcoxon-Ranksum test. Bivariate and multivariate logistic regression analyses were conducted to establish associations and explore relationship between covariates and HIV transmission.

**Results:**

One thousand and one hundred nineteen (42.4%) infants had dried blood spot samples taken for HIV deoxyribonucleic acid polymerase chain reaction test within the first 6 weeks of age. Median age at diagnosis for HIV-positive infants was 4 months (IQR 1.5–9) while that of HIV-negative infants was 2 months (IQR 1.5–6). In total, 1,906 (72.1%) infants received prophylactic antiretrovirals. Infants whose mothers received prophylaxis had significantly lower vertical transmission rate (6.7%) compared to those whose mothers did not receive prophylaxis (24.0%), (OR 0.23, *p* < 0.001). When adjusted for feeding option and infant’s age at diagnosis, the odds of transmission among women who received prophylaxis was 76% lower than that of women who did not receive any prophylaxis (OR 0.2 *p* < 0.001). 1,368 infants less than 6 months of age, 67.3%) were exclusively breastfed, 214 (10.5%) were replacement fed, and 164 (8.1%) mixed fed. Mixed feeding was associated with increased risk of HIV transmission (OR 2.7, *p* = 0.007). 67% of children older than 6 months were breastfed and had higher HIV transmission rate compared to those who were not breastfed (OR 2.3, *p* = 0.006).

**Conclusions:**

The recorded rate of 9.3%, suggest the interventions implemented at the study sites were moderately effective, more so when provided early. Program performance will improve should the 12.8% of pregnant women who did not receive antiretroviral prophylaxis are reached.

## Background

In 2011, the United Nations General Assembly committed to the Global Plan that sought to eliminate new HIV infections among children by 2015 and keep their mothers alive [1]. Subsequently, the World Health Organization (WHO) issued the consolidated guidelines in 2013, recommending lifelong use of antiretroviral therapy (ART) for all pregnant women regardless of their CD4 count [2, 3]. This intervention is also referred to as prevention of mother-to-child transmission (PMTCT) option B+. However, despite its proven efficacy and cost effectiveness, the adoption of PMTCT Option B+ remains slow [3–5].

Numerous contextual and health systems factors hamper the adoption of best practices in resource limited settings. In Sub-Saharan Africa where 90% of mother-to-child transmission (MTCT) of HIV occurs, achieving 90% reduction of MTCT (from 2009 estimates) and reaching a transmission rate of ˂ 5% by 2015 remains a challenge [6]. It has been established that women and infants who do not access PMTCT intervention have 15–30% risk of MTCT in pregnancy and delivery, which increases to 20–45% during breastfeeding [7]. Early Infant Diagnosis (EID) remains a key test in measuring the extent to which PMTCT interventions have averted MTCT. It is also the entry point for HIV-exposed infants (HEI) to early clinical evaluation, prophylaxis for opportunistic infections and ART [3]. In resource limited settings, without any therapeutic intervention, up to 35 and 52% of HIV infected infants die by age one year and two years respectively [7, 8].

Although several studies have been done in Sub-Saharan Africa on the effectiveness of PMTCT programs and factors associated with HIV sero-positivity among HIV exposed infants [9–11], little is known of the effectiveness of the PMTCT programs in the Kenyan public health sector context. The successful roll out of proven effective public health interventions such as PMTC is highly dependent on the ability of country health systems to embed the interventions in routine service delivery and people in need must be able to utilize the services. The purpose of our study was to assess the effectiveness of a PMTCT program implemented in the South Rift region of Kenya. The measurement of vertical transmission rates of HIV in a program serves as proxy of the degree of availability and uptake of PMTCT services. This information is critical to inform public health policy, planning and resource allocation.

## Methods

### Study design

This was a cross-sectional survey of the EID results of infants enrolled in the USAID-funded APHIA Plus project in the South Rift region of Kenya between January 1^st^ and December 31^st^ 2013. The survey included the EID records of all HIV exposed infants enrolled in the AIDS, Population and Health Integrated Assistance (APHIA) Plus Rift project-supported health facilities.

### Setting and sample

The sites included in this study were public health facilities that cater mostly for uninsured patients, majority of whom were of low socio economic status. The South Rift region is predominantly rural and consists of five counties namely; Baringo, Kajiado, Laikipia, Nakuru and Narok, with a total population of 4,888,222 in 2013. Agriculture is the mainstay of the economy. The APHIA Plus project supported health facilities in all the five counties. The majority (70%) were primary care facilities [12]. The support to PMTCT sites consisted of skills building through mentorship, orientation to new guidelines, standard operating procedures and job aids; sensitization to best practices and on the job training; laboratory networking for CD4, PCR and viral load samples referral; support to mentor mothers and sessional counsellors.

In total, 2772 dry blood spot (DBS) were tested during the observation period. Using both sample code and facility name, 94 duplicate entries were identified and removed. Further data cleaning excluded 35 records whose HIV status was missing and one record whose value for infant age was above 18 months at the time of diagnosis. After data cleaning our final data set consisted of 2642 records. The majority of records were from Nakuru (1591, 60.2%), followed by Kajiado (499, 18.9%), Narok (222, 8.4%), Laikipia (189, 7.2%), and Baringo 141 (5.3%).

### Procedure

Typically, HIV counselling and testing on an opt-out approach was provided through a trained health care worker to expectant mothers on their first visit as part of the antenatal care package. Nationally approved testing algorithms were used (Determine® as the first test, Bio-line® as the second test, and Unigold® as the tie-breaker). For consenting mothers, results were provided the same day [13]. Women who tested negative for HIV were further counselled on prevention. Women who tested positive were started on antiretroviral prophylaxis as per the prevailing Kenyan guidelines that gave clinicians four options: i) single dose Nevirapine at onset of labor; ii) maternal AZT from 14 weeks gestation with addition of 3TC and Nevirapine at onset of labor, and infant AZT and 3TC for a week; iii) ART during pregnancy and cessation after breastfeeding (interrupted ART) and iv) lifelong ART initiated at first contact with the mother during pregnancy (Option B+) [14]. HIV positive pregnant women were enrolled in care, and given a comprehensive package as stipulated in the national PMTCT guidelines. Upon birth the infants were also enrolled in a follow up program in the maternal, newborn and child health unit [14].

EID in APHIAPlus Rift supported facilities followed the national algorithm. To establish HIV exposure status, all infants of unknown status aged ≤18 months received an HIV antibody test at their first clinical visit. Infants who turned positive on HIV antibody test were referred for a DNA PCR test. Using the heel stick method, DNA PCR samples were obtained as DBS on Whatman filter paper and shipped to one of the six reference laboratories as determined by both proximity and ease of transport logistics. The six reference laboratories were: i) Kenya Medical Research Institute CVR HIV-P3 in Nairobi County; ii) Kenya Medical Research Institute CDC HIV/R in Kisumu; iii) Kenya Medical Research Institute CIPDCR Alupe in Busia County; iv) Kenya Medical Research Institute/Walter Reed CRC in Kericho County; v) AMPATH reference laboratory Eldoret in Uasin Gishu County; and vi) Coast provincial general hospital molecular laboratory in Mombasa county.

DNA PCR positive infants were started on co-trimoxazole and ART from the age of 6 weeks. DNA PCR negative infants were retested at 9 months. A confirmatory DNA PCR test was offered to HIV antibody positive infants. DNA PCR negative infants received an HIV antibody repeat test at 18 months and if older, a week after cessation of breastfeeding.

DBS samples collected from HIV exposed infants in APHIA Plus-Rift supported facilities were transported to one of six national reference laboratories. Once processed and analyzed, results were transmitted back to the referring health facility in both hard and digital copy in sites with an existing SMS printer. In addition, the national reference laboratory retained records of all test done and reported into the national EID database accessible through the National AIDS and STI Control Program website [15]. The website has inbuilt validation checks that serve to minimize errors during data entry. We accessed these routine service data for the APHIA Plus -Rift supported sites from the NASCOP’s EID website, downloaded as an excel spreadsheet.

### Variables

The standard EID data collection tool has fields for maternal and infant data. Key variables considered included: demographic data; type of maternal antiretroviral prophylaxis regimen received; infant feeding options adopted; and facility-entry point/department at diagnosis and infants’ age at testing. Infant feeding options were classified as exclusive breastfeeding, mixed, and replacement feeding for infants under-six months; and breastfeeding and no breastfeeding for those above six months at the time of testing.

Facilities were categorized according to the Kenya Health Policy into tier one (community level), tier two (primary level), tier three (secondary or county level referral hospital) and tier four (tertiary level referral facilities) [12].

### Statistical analysis

Data was exported into STATA/SE 12.1 (STATA Corporation LP 4905 Lakeway Drive) for analysis. Our primary outcome of interest was the DBS test result as an indicator of PMTCT interventions success or lack thereof. We compared the characteristics of HIV exposed infants who tested positive to those who tested negative. We used the Chi-square test for categorical variables and Wilcoxon-Ranksum test and median tests for age variable which followed a non-normal distribution. In records where the feeding option was considered invalid based on the national EID reporting tool, the value was set to missing. We conducted bivariate logistic regression analysis to examine associations between the dependent variable (infant HIV status) and independent variables such as maternal antiretroviral regimen, infant’s age at DBS test, infants’ sex, the method of feeding and the health facility category (tier). We included variables that had a *p* value of ≤ 0.25 in the bivariate analyses.

We then conducted multivariable logistic regression to explore relationship between various covariates and vertical HIV transmission. We also explored effects of confounders and interactions of various covariates on vertical transmission.

## Results

### Description of infants in the study

Infant age in the dataset ranged from birth to 18 months distributed as follows: 42% aged 6 weeks and below, 48% aged between 6 weeks and 9 months, 9% were above 9 months and only 1% were missing age variable. The majority (77.4%) were aged less than 6 months. A hundred and twelve records (4.2%) had no sex recorded. (Table 1).

**Table 1:** Baseline Characteristics of infants in the study (*N* = 2642)

Characteristics	N = 2642	EID test outcome (HIV status)	
	Frequency	EID test positive	EID test negative
		n (%)	n (%)
Sex			
Male	1271	128 (10.1)	1143 (89.9)
Female	1259	106 (8.4)	1153 (91.6)
Missing	112	11 (9.8)	101 (91.2)
Age at testing			
≤ 6 weeks	1119	71 (6.3)	1048 (93.7)
> 6 weeks to 9 months	1261	115 (9.1)	1146 (90.9)
> 9 months	244	58 (23.8)	186 (76.2)
Missing	18	1 (5.6)	17 (94.4)

Overall 245 infants tested positive. This represents a 9.3% positivity rate. Only 1,119 (42.4%) of the infants were tested within the first 6 weeks of age as per the national guidelines while 1,261 (47.7%) were tested between 6 weeks and 9 months. Two hundred and forty-four (9.2%) infants were tested after 9 months. Overall, the median age at the time of DBS sample collection was 2 months (Interquartile range (IQR); 1.5 – 6 months). HIV positive infants however were significantly older at the time of first DBS sample collection (OR 1.1[1.07–1.13] *p* < 0.001) compared to those who tested negative. The median age at diagnosis for the positive infants was 4 months (IQR 1.5 – 9 months), double the median age for the negative children (2 months; IQR 1.5 – 6 months).

### Uptake of maternal antiretroviral prophylaxis and infant feeding practices

Table 2 shows that almost three quarters of infants’ mothers 1,906 (72.1%) received ARV for PMTCT. Out of these, 810 (42.5%) received the more efficacious life-long ART regime and 133 (7.0%) were on interrupted ART. The rest received either Zidovudine from 14 weeks (32.3%), single dose Nevirapine (2.7%) or other unspecified regimens (15.5%). About two thirds of infants < 6 months of age (1368, 67.3%) were exclusively breast-fed, 214 (10.5%) were on replacement feeding and 164 (8.1%) were on mixed feeding. Data on feeding option were missing in 286 (14.1%) entries. More than half the infants aged ***>***6 months (357, 60.3%) were still breast feeding. A total of 245 (9.3%) HIV exposed infants tested positive with no significant difference between male (10.1%) and female (8.4%) (OR 1.22, *p* = 0.152).

**Table 2:** Maternal antiretroviral prophylaxis and infant feeding options among the infants

Uptake of maternal antiretroviral prophylaxis	*n* = 2642, (%)	Positive n(%)	Negative n(%)
Received prophylaxis	1906 (72.1)	127 (6.7)	1779 (93.3)
No prophylaxis	338 (12.8)	81 (24.0)	257 (76.0)
Missing	398 (15.1)	37 (9.3)	361 (90.7)
Type of maternal antiretroviral regimen	*n* = 1906, (%)		
Lifelong ART	810 (42.5)	48 (5.9)	762 (94.1)
AZT + (3TC + sd-NVP at labour) + (AZT + 3TC for 7 days)	616 (32.3)	41 (6.7)	575 (93.3)
Interrupted ART	133 (7.0)	6 (4.5)	127 (95.5)
Sd-NVP only	51 (2.7)	1 (2.0)	50 (98.0)
Others	296 (15.5)	31 (10.5)	265 (89.5)
Infant feeding option			
< 6 months	*n* = 2032, (%)		
Exclusive replacement feeding (ERF)	214 (10.5)	12 (5.6)	202 (94.4)
Exclusive breastfeeding (EBF)	1368 (67.3)	97 (7.1)	1271 (92.9)
Mixed feeding (MF)	164 (8.1)	23 (14.0)	141 (86.0)
Missing	286 (14.1)	22 (7.7)	264 (92.3)
> 6 months	*n* = 592, (%)		
Not breastfeeding (NBF)	179 (30.2)	16 (8.9)	163 (91.1)
Breastfeeding (BF)	357 (60.3)	65 (18.2)	292 (81.8)
Missing	56 (9.5)	9(16.1)	47 (83.9)

Although there was no significant variation in transmission rates between facility tiers (primary care, county or national referral) we observed this variation between counties. Narok County had a significantly higher positivity compared to Baringo county which had the lowest positivity (OR 2.99, *p* = 0.012). (Table 3).

**Table 3:** Univariate analysis of infant HIV status by health facility tier and by county

Facility Tier	*N* = 2642	EID test outcome (HIV status)		OR [95% CI]	*p* -value
	Total	Negative n(%)	Positive n(%)		
Tier 2	1053	963 (91.4)	90 (8.6)	Ref (1)	
Tier 3	1125	1015 (90.2)	110 (9.8)	1.16 [0.87–1.6]	0.32*
Tier 4	464	419 (90.3)	45 (9.7)	1.15 [0.79–1.67]	0.47*
County name	*N* = 2642				
Baringo	141	134 (95.0)	7 (5.0)	Ref.	(Ref)
Kajiado	499	457 (91.6)	42 (8.4)	1.76 [0.77–4.0]	0.18*
Laikipia	189	157 (91.0)	17 (9.0)	1.89 [0.76–4.69]	0.17*
Nakuru	1591	1442 (90.6)	149 (9.4)	1.98 [0.91–4.31]	0.09*
Narok	222	192 (86.5)	30 (13.5)	2.99 [1.28–7.01]	0.01**

*OR[95% CI]* unadjusted Odds ratio with the 95% confidence interval, *Ref (1)* Reference category

*: Statistically insignificant (*P* ≥ 0.05), **: Statistically significant (*P* < 0.05)

### Determinants of infant's HIV

From bivariate analyses, infants whose mothers received antiretroviral prophylaxis had a significantly lower HIV vertical transmission rate (6.7%) compared to infants whose mothers did not receive prophylaxis (24.0%) (OR 0.23; *p* < 0.001). The type of antiretroviral regimen taken by the mother was significantly associated with a negative DBS test result (*p* <0.001). Mixed feeding was associated with close to threefold increase in the risk of vertical transmission among infants aged less than six months (OR 2.75; *p* =0.007). Continued breastfeeding alongside complementary feeds beyond six months was associated with increased transmission rates (OR 2.27; *p* =0.006). See Table 2 above.

In our adjusted model, we found that all antiretroviral regimens used perinatally reduced the rates of vertical HIV transmission. ART for instance, reduced vertical HIV transmission rates by 70% (aOR 0.3 *p* < 0.001) while AZT combined with single dose nevirapine and 3TC reduced transmission by 59% (aOR 0.41, *p* <0.001). There was no statistically significant difference in transmission rates between children whose mothers were on ART and those on AZT + single-dose nevirapine + 3TC (aOR 0.74 *p* = 0.211). Compared to infants enrolled through MCH, the odds of a positive DNA PCR result for infants enrolled through other entry points was 1.44 (*p* < 0.001). Children who were enrolled through the inpatient wards were significantly more likely to be HIV-infected compared to those enrolled through the MCH (aOR 5.13, *p* < 0.001). There was no significant difference in transmission rates between the five counties in the adjusted model.

Although age at DNA PCR test and the mode of infant feeding were found to be significant in our bivariate analyses, in multivariable model these two variables ceased to be significant. The infant’s sex was not significantly associated with the outcome in both bivariate and multivariable analyses (see Table 4)

**Table 4:** Determinants of the positivity of HIV exposed infant’s HIV test

Variable name	OR [95% CI]	*p* value	OR [95% CI]	*p v*alue
Infant age at diagnosis *n* = 2624				
i. ≤ 6 weeks	1 (Ref)		1 (Ref)	
ii. >6 weeks – 9 Months	1.48 [1.09–2.01]	0.012**	1.54 [1.03–2.31]	0.037**
iii. >9 Months	4.60 [3.15–6.73]	<0.001**	3.61 [1.80–7.23]	<0.001**
Infants’ sex				
Female	1 (Ref)			
Male	1.22 [0.93–1.60]	0.152*	1.16 [0.84**–**1.59]	0.368*
Maternal prophylaxis *n* = 2244				
Any form of antiretroviral prophylaxis	1 (Ref)		1(Ref)	
No antiretroviral prophylaxis	4.4 [3.2–6.0]	<0.001**	2.9 [2.0–4.1]	<0.001**
Maternal antiretroviral regimen (type) *n* = 2244				
i. None (did not receive ARVs)	1 (Ref)		1(Ref)	
ii. HAART	0.20 [0.14–0.29]	<0.001**	0.30 [0.19–0.47]	<0.001**
iii. Interrupted HAART	0.15 [0.06–0.35]	<0.001**	0.27 [0.11–0.67]	0.003**
iv. AZT + (3TC + sd-NVP at labour) + (AZT + 3TC for 7 days)	0.23 [0.15–0.34]	<0.001**	0.41 [0.26–0.65]	<0.001**
v. SdNVP	0.06 [0.01–0.47]	0.007**	1	
vi. Others	0.37 [0.24–0.58]	<0.001**	0.49 [0.29–0.82]	0.005**
Mode of infant feeding				
Infants ≤ 6 months n=				
Exclusive replacement feeding	1 (Ref)		1 (Ref)	
Exclusive breastfeeding	1.28 [0.69–2.38]	0.4*	1.27 [0.68–2.38]	0.456*
Mixed feeding	2.75 [1.32–5.70]	0.007**	1.73[0.81–3.7]	0.158*
Infants >6 months				
Not breastfed	1 (Ref)			
Breast fed	2.3 [1.3–4.0]	0.006**	1.87 [1.02–3.44]	0.043**

*Ref* reference category, *OR* Odds ratio, *aOR* Adjusted Odds ratio

* = Not significant (*p* > 0.05), ** = Significant (*p* <0.05), [95% CI] : 95% Confidence interval

## Discussion

The PMTCT program in the APHIAPlus Rift project may have been effective as evidenced by the results of lower transmission rates (9.27% overall positivity) among the population attending PMTCT programs, compared to15-45% transmission rates expected from literature with no PMTCT intervention. This however falls short of the target of reducing MTCT rates to less than 5%. Although 72% of mothers in the study population receiving any form of antiretroviral prophylaxis, access to the more efficacious regimes including the option B+ remains low (53%) and only 42% of HEI test at ≤6 weeks of age. Yet our study findings suggest early infant age at PCR testing, exclusive breastfeeding and lifelong use of maternal ART for prophylaxis are associated with lowered vertical transmission of HIV.

### Timing of early infant diagnosis

The 2013 WHO guidelines call for the first DNA PCR for early infant diagnosis at between 0 and 6 weeks [3]. This mirrors the Kenyan guidelines which make a similar recommendation of conducting the first DNA PCR at 0–6 weeks, In our study 1119 (42.4%) of infants received their first DNA PCR at between 0 and 6 weeks. In earlier multi-country analyses of Nigeria, Uganda and Namibia, the proportion of first DNA PCR tests done in the first two months of life was less than 50% [16]. The lowest ages were seen at tertiary centers that were known to have robust PMTCT services [16, 17]. Although the latter examined infants up to the age of two months, we project that if we examined a similar group, our uptake of the first DNA PCR would be slightly higher. We do note however that the multi-country analyses were done in 2011; 2 years earlier than our study hence the divergent comparison group – under two months vis-a-vis 0–6 weeks. That notwithstanding, our results may reflect the growing impetus towards earlier testing, a move considered pivotal in curbing perinatal infections [18].

Further, we found that on DNA PCR testing, infants who tested positive were older than those who tested negative. This is consistent with current findings in Kenya. Hassan et al. noted that late entry at enrollment into EID program and high dropout rate were associated with increased rates of positivity. Majority of infants enrolled at over two months of age, had been referred from acute or chronic clinical services and therefore sick at the outset [19]. Elsewhere, findings from the Kilimanjaro region of Tanzania are no different [20]. O’Donnell et al. observed that reluctance to child testing may be related to various issues around acceptability. Cultural norms dictating that young infants should not be taken outside the house except for receipt of vaccine (not always collocated with EID), serve to provide insights into this observation [20].

### Infant feeding options

In our study, over two thirds 1368 (67.3%) of infants younger than six months, were breastfed exclusively; three fifths, 357 (60.3%) of infants above 9 months were breastfeeding when the first DNA PCR was taken. Similar high rates of exclusive breastfeeding were observed in Nigeria and Zambia at 80 and 84% respectively [21, 22]. Findings of high exclusive breastfeeding rates in the general population are uncommon and therefore surprising since adherence to exclusive breastfeeding in our setting is generally low [23]. In many societies of sub-Saharan Africa, encouraging mothers to practice exclusive breastfeeding is far from easy. Often, babies are given water, tea, porridge or other foods alongside breast milk in the first few weeks of life [24]. A study done in Malawi to find out about feeding practices of children showed that 65% of infants were given other foods within 1 month of life and only 4% of infants were exclusively breastfed [25]. In Cameroon, Noubiap et al. reported exclusive breastfeeding rates of 44.6%, while Tejiokem et al. found exclusive breastfeeding rates of 10.7% [17, 26]. According to the KDHS 2008–09, exclusive breastfeeding is not common as only 32% of infants less than 6 months are exclusively breastfed [23]. Additionally, infants in our study who had been categorized as either having been breastfed exclusively or mixed fed showed an incremental transmission rate with age.

We found a third (32.4%) of mothers received the option B_+_, while slightly over a fifth (22.9%). received AZT mono-therapy and single-dose Nevirapine at onset of labor. In line with evidence elsewhere, the use of maternal prenatal antiretroviral prophylaxis was significantly associated with a lower vertical HIV transmission rate (unadjusted OR 0.23 *P* < 0.001) [21, 22, 27]. However, in Sub-Saharan Africa maternal access to ARVs remains sub-optimal [2]. In our study, vertical transmission rate was 3.6 times higher among the 320 (14.8%) infants whose mothers did not access prenatal prophylactic ARVs compared to infants whose mothers used prophylactic ARVs.

### Type of PMTCT antiretroviral regimen

Currently, the WHO recommended Option B plus – triple ART is preferred as the most cost effective regimen at stemming vertical HIV transmission [3, 5]. Kenya recently adopted the WHO recommendations during the last quarter of 2014, and there is already a significant increase in the proportion of the mothers initiated on ART as compared to option A. Our findings are consistent with evolution of recommendations on maternal antiretroviral regime use both nationally and internationally. With dwindling use of single dose Nevirapine (1.7%), our findings reflect positive adoption of PMTCT guidelines and best practices over the years.

On further analysis, when we adjusted for feeding option and infant’s age at diagnosis, the odds of vertical transmission among women on HAART was 79% lower that of single-dose Nevirapine (aOR 0.21 *P* < 0.001). We found no significant difference in transmission rates between ART and use of AZT plus peripartum sdNVP -option A (*p* = 0.211). Our findings are consistent with those of the Kesho Bora trial that compared extended maternal triple antiretroviral prophylaxis through 6 months of breastfeeding with prenatal AZT/sd-NVP without a postpartum component and found no significant difference [28]. Although data continue to show equal efficacy between Option A and option B, use of life-long triple antiretroviral therapy provides additional benefits of a slowed HIV maternal progression and averting sexual transmission to sero-discordant couples [3, 5].

### Methodological considerations

Our study has limitations related to missing data, as is common when using routine service statistics. Furthermore, the unavailability of data on maternal viral load and peripartum factors made it impossible for our study to account for the role of these variables on vertical transmission of HIV. However, an ad hoc analysis of missing data revealed no significant difference in characteristics. In the end, we restricted our analysis to records with no missing data. We note that most infants (67.3%) were reportedly to have been exclusively breastfed. Whilst this is a desired practice, this high proportion could be attributed to social desirability bias. Since this was a self-report, most women would report as expected by health care providers’ i.e. exclusive breastfeeding for HIV exposed infants. Our results may not be generalizable beyond the South Rift region owing to the purposeful selection of the facilities. Only APHIAPlus-supported facilities were considered in this study. Despite these limitations, findings from this study are still useful to policy makers and program managers for continued improvement in the EID program.

## Conclusion

PMTCT interventions supported by the APHIAPlus Rift project were moderately effective in reducing MTCT as the target of reaching transmission rates of <5% was not achieved. Early infant age at PCR testing, exclusive breastfeeding for infants less than 6 months of age, and lifelong use of maternal ART for prophylaxis are associated with lowered vertical transmission of HIV. It is possible to reach the target of MTCT rates of < 5% in Kenya provided more be done to close the gap for those women and infants attending the PMTCT sites yet do not access PMTCT interventions and increase the proportion of those receiving the more efficacious antiretroviral prophylaxis regimen. To this end, additional quality improvement and health systems strengthening interventions are recommended.

## Abbreviations

3TC: Lamivudine
APHIA: AIDS, population and health integrated assistance
ART: Antiretroviral therapy
AZT: Zidovudine
CD4: Cluster of differentiation 4
DBS: Dry blood spot
DNA: Deoxyribonucleic acid
EID: Early infant diagnosis
HIV: Human immunodeficiency virus
IQR: Interquartile range
MTCT: Mother to child transmission of HIV
NASCOP: National AIDS/STD Control Program
OR: Odds ratio
PCR: Polymerase chain reaction
PMTCT: Prevention of mother to child transmission of HIV
WHO: World Health Organization
